# Path instability of an air bubble rising in water

**DOI:** 10.1073/pnas.2216830120

**Published:** 2023-01-17

**Authors:** Miguel A. Herrada, Jens G. Eggers

**Affiliations:** ^a^Escuela Técnica Superior de Ingeniería, Universidad de Sevilla, Seville 41092, Spain; ^b^School of Mathematics, University of Bristol, Bristol BS8 1UG, United Kingdom

**Keywords:** bubbles, hydrodynamic stability, numerical methods, boundary layers

## Abstract

It has been documented since the Renaissance that an air bubble rising in water will deviate from its straight, steady path to perform a periodic zigzag or spiral motion once the bubble is above a critical size. Yet, unsteady bubble rise has resisted quantitative description, and the physical mechanism remains in dispute. Using a numerical mapping technique, we for the first time find quantitative agreement with high-precision measurements of the instability. Our linear stability analysis shows that the straight path of an air bubble in water becomes unstable to a periodic perturbation (a Hopf bifurcation) above a critical spherical radius of *R* = 0.926 mm, within 2% of the experimental value. While it was previously believed that the bubble’s wake becomes unstable, we now demonstrate a new mechanism, based on the interplay between flow and bubble deformation.

The motion of bubbles in water plays a central role for a wide range of natural phenomena, from the chemical industry to the environment (1). The buoyant rise of a single bubble serves as a much-studied paradigm, both experimentally (2–4) and theoretically (2, 5–9). Yet, in spite of these efforts, and in spite of the ready availability of enormous computing power, it has not been possible to reconcile experiments with numerical simulations of the full hydrodynamic equations for a deformable air bubble in water. This is true in particular for the intriguing observation, made already by Leonardo da Vinci (10, 11), that sufficiently large air bubbles perform a periodic motion, instead of rising along a straight line.

Indeed, a rising air bubble presents great numerical and theoretical challenges. First, the small viscosity of water implies the appearance of thin boundary layers (12), which have to be resolved accurately in order to capture the interplay between buoyancy and dissipation, which sets the speed of rise. Second, the ability of the exterior fluid to glide over the bubble surface without friction (the effect of the gas can safely be neglected) means that viscous effects arise only on account of flow line curvature, whose subtle effect has to be captured accurately. Third, and most significantly, the bubble deforms in response to the forces exerted by the fluid, and in turn, the shape of the bubble changes the character of the flow.

The vanishing resistance to shear on the bubble surface also means that experiments are extremely sensitive to contamination by surfactants (1–3, 13), which partially mimic the no-slip boundary conditions of a solid particle. As a result, experimental rise velocities as well as critical bubble sizes have proved inconsistent (14). We base our comparison on the seminal experiments of Duineveld (3, 13), using “hyper clean” water and whose data for the terminal bubble speed *V*_*t*_ is shown as circles in Fig. 1*A*. The solid line is the result of our numerical simulations we describe now.

**Fig. 1.**
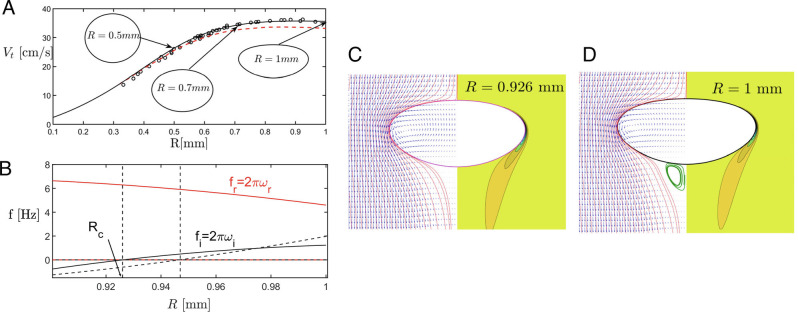
(*A*) The rise speed *V*_*t*_ as function of the undeformed bubble radius *R*; the symbols are the data of (3), the solid line shows the simulation with *ρ* = 998.3 kg/m^3^, *μ* = 1.014 mPa/s, *γ* = 72.8 mN/m; the red dashed line is the theory of (5); bubble shapes as insets. (*B*) Real and imaginary parts of the frequency 2*πω* near the transition; first eigenvalue (largest imaginary part): black and red solid lines, second eigenvalue: dashed lines; *ω*_*i*_ = 0: vertical dashed lines. (*C* and *D*) The flow field at *R*_*c*_ = 0.926 mm and *R* = 1 mm. On the left, red flow lines and blue arrows to represent the flow velocity. In (*D*), recirculating flow lines at the rear are shown in green. On the right, azimuthal vorticity contours.

## Numerical Simulations

Our simulations use the Navier-Stokes equations (12) for two incompressible ($\nabla \cdot v_{i}=0$) fluids, water, and air (i = l,g):

[1] $$\begin{array}{c} \frac{\partial v_{i}}{\partial t}+(v_{i}\cdot \nabla )v_{i}=-\frac{\nabla p}{\rho_{i}}+\frac{\mu_{i}}{\rho_{i}}▵v_{i}-(g+\frac{\mathrm{dV}}{\mathrm{dt}})e_{z}, \end{array}$$

with standard values for density *ρ* and viscosity *μ*, *g* is the acceleration of gravity; (*z*, *r*, *θ*) is a cylindrical coordinate system, **v** = (*w*, *u*, *v*) is the velocity, and *p* is the pressure. We use a noninertial frame of reference, in which the top of the bubble is stationary; *V* is the vertical speed of the top of the bubble in the laboratory frame, which is constant for steady rise, but accelerating in the unsteady regime.

The free surface is parameterized as *r* = *f*(*s*, *θ*, *t*) and *z* = *h*(*s*, *θ*, *t*), where *s* is the meridional arclength (0 ≤ *s* ≤ 1); *f* and *h* are computed from the fluid velocity using the kinematic boundary condition. Liquid and gas velocities are continuous across the bubble surface, where boundary conditions are $(\sigma_{l}-\sigma_{g})\cdot n=\gamma \kappa n$, $\sigma =-pI+\mu (\nabla v+\nabla v^{T})$ the stress tensor; here, *γ* is the air–water surface tension, and *κ* (twice) is the mean curvature. An equation for *V* is obtained by requiring that on the top *s* = 1 of the bubble, *w* = 0, *f* = 0, and the height at the top is held constant at *h* = *R*. The entire fluid domain is enclosed in a large sphere of radius *R*_*out*_ ≫ *R*; *SI Appendix* for details of the boundary conditions on the outer sphere.

The key idea of the numerics is to map the physical domain such that the free surface becomes a fixed rectangle (15), obviating the need to track the interface. The liquid domain can be described in closed form as a mapping

[2] $$\begin{array}{cc} r & =f\left(s,\theta ,t\right)+(R_{\mathrm{out}}\sin\left(\pi s\right)-f\left(s,\theta ,t\right))\eta ,\\ z & =h\left(s,\theta ,t\right)+(R_{\mathrm{out}}\cos\left(\pi s\right)-h\left(s,\theta ,t\right))\eta , \end{array}$$

such that *η* = 0 is the free surface and *η* = 1 is the outer sphere; inner mapping: *SI Appendix*. The entire problem of writing *w*_*l*_, *u*_*l*_, *v*_*l*_, *p*_*l*_ and *V* as a function of *s*, *η*, *θ*, and *t* can now be discretized using standard methods; points are concentrated near the bubble surface to resolve boundary layers. Eq. **2** implies that analytical expressions for the derivatives become extremely complicated, but they are calculated automatically using MATLAB’s “Symbolic Math Toolbox” package and saved using the “matlabFunction” routine.

### Base State.

We solved the nonlinear equations for steady, axisymmetric bubble shapes using a Newton scheme. In Fig. 1*A*, the speed of rise *V*_*t*_ (solid line) is seen to be in excellent agreement with the experiment, except for the smallest bubbles; similar agreement was achieved comparing to the data of Sanada et al. (4). The entire numerical data set takes less than a day to compute on a personal workstation. The dashed line is Moore’s asymptotic theory for small bubbles (5), which approximates the bubble shape as an ellipsoid. While there is perfect agreement for *R* ≲ 0.4 mm, the theory gradually falls below the result of the simulations. Indeed (Fig. 1*A*), with increasing *R*, the bubble shape increasingly loses its up–down symmetry: it becomes flat on the top and rounded at the bottom.

The structure of the flow around the bubble is seen in Fig. 1 *C* and *D*; on the left, flow lines follow the bubble surface closely, similar to the solution of the potential flow problem; only for *R* near 1 mm does a small recirculating region appear at the rear of the bubble. This agrees with time-dependent numerical simulations (9), which for very small Morton numbers (Mo = *gμ*^4^/(*ργ*^3^) = 2.63⋅10^−11^ in our case) find no standing eddy near the transition toward time-dependent motion. In case of vanishing tangential stress, vorticity is generated on the surface in proportion to the curvature (12), which is concentrated around the equator and is then shed downstream (Fig. 1 *C* and *D*, *Right*). The theoretical calculation of the speed of rise is based on the dissipation in the potential flow solution, with a correction coming from the thin boundary layer in which vorticity is concentrated (5). Increased surface vorticity reduces the flow speed on the surface, making velocity gradients weaker, reducing dissipation, so that the bubble rises more quickly (1).

### Linear Stability.

With *Ψ*_*b*_(*z*, *r*) being any one of the dependent variables describing the bubble’s steady, axisymmetric rise, we study stability to general three-dimensional (3D), time dependent perturbations:

[3] $$\begin{array}{c} \Psi \left(z,r,\theta ;t\right)=\Psi_{b}\left(z,r\right)+\epsilon \;\delta \Psi \left(z,r\right)e^{-i\omega t+im\theta }, \end{array}$$

where *m* = 0, ±1, ±2, …. Inserting Eq. **3** into the equations of motion and linearizing in *ϵ*, for each *m* and fixed unperturbed bubble radius *R*, we obtain an eigenvalue problem, with eigenvalue *ω* = *ω*_*r*_ + *iω*_*i*_ and eigenfunction *δΨ*(*z*, *r*). For a given *m*, we are interested in the value of *R* for which *ω*_*i*_ first becomes positive, corresponding to exponential growth; modes *m* = ±1 are the most unstable, so we focus on them for the remainder.

Eigenvalues and eigenfunctions are found from solving the generalized eigenvalue problem

[4] $$\begin{array}{c} J_{b}^{\left(p,q\right)}\delta \Psi^{\left(q\right)}=i\omega Q_{b}^{\left(p,q\right)}\delta \Psi^{\left(q\right)}; \end{array}$$

analytical expressions for the Jacobians 𝒥_*b*_^(*p*, *q*)^ and 𝒬_*b*_^(*p*, *q*)^ are once more calculated in MATLAB; Eq. **4** is solved in MATLAB. One shows (7) that if *Ψ* is identified as *v*_*z*_, *v*_*r*_, and *imv*_*θ*_, Eq. **4** depends on *m*^2^ only, and matrices are real. Thus, eigenvalues and eigenfunctions appear in complex conjugate pairs *ω* = ±*ω*_*r*_ + *iω*_*i*_ and are the same for *m* = ±1.

In Fig. 1*B*, *ω*_*i*_, *ω*_*r*_ are shown for *R* between 0.9 mm and 1 mm. The first eigenvalue to become unstable (with *ω*_*i*_ turning positive at *R*_*c*_ = 0.926 mm, vertical dashed line) is shown with solid lines, agreeing very well with the critical bubble radius of *R* = 0.91 mm reported in ref. (3). This occurs at a finite value of *f* = 2*πω*_*r*_ ≈ 6.3 Hz (a Hopf bifurcation), consistent with the experimental value of *f* = 6.4 Hz at *R* = 1.15 mm (13). The next smaller imaginary part only passes through zero at *R* = 0.94 mm, with vanishing real part, causing no oscillation. Previous studies (7, 8, 16), which make the assumption of a fixed ellipsoidal shape, have found the opposite order of eigenvalues. Hence for a correct description of the instability, the interplay between shape deformations and flow needs to be considered.

## Mechanism of Instability

Time-dependent solutions can be constructed as linear combinations of modes *a*_1/2_*δΨe*^−*iω*_*r*_*t* ± *iθ*^, *a*_3/4_δΨ^*^e^*iω*_*r*_*t* ± *iθ*^ in Eq. **3**, such that the result is real. At the Hopf bifurcation (*R* = *R*_*c*_), only a single mode is unstable. Choosing *a*_1_ = *a*_4_ real and *a*_2_ = *a*_3_ = 0, we obtain *δΨ* = 2*a*_1_(*δΨ*_*r*_ cos(*θ*−*ω*_*r*_*t*)−*δΨ*_*i*_ sin(*θ*−*ω*_*r*_*t*))*e*^*ω*_*i*_*t*^, corresponding to a bubble turning clockwise, where *δΨ* = *δΨ*_*r*_ + *iδΨ*_*i*_. If on the other hand, *a*_1_ = *a*_2_ real and *a*_3_ = *a*_4_ = 0, *δΨ* = 4*a*_1_ cos*θ*[*δψ*_*r*_ cos(*ω*_*r*_*t*)+*δψ*_*i*_sin(*ω*_*r*_*t*)]*e*^*ω*_*i*_*t*^, describing a zigzag motion in one plane. Thus, which motion is observed experimentally depends on the initial conditions or is selected by nonlinear effects. For simplicity, we will assume zigzag motion in the following.

**Fig. 2.**
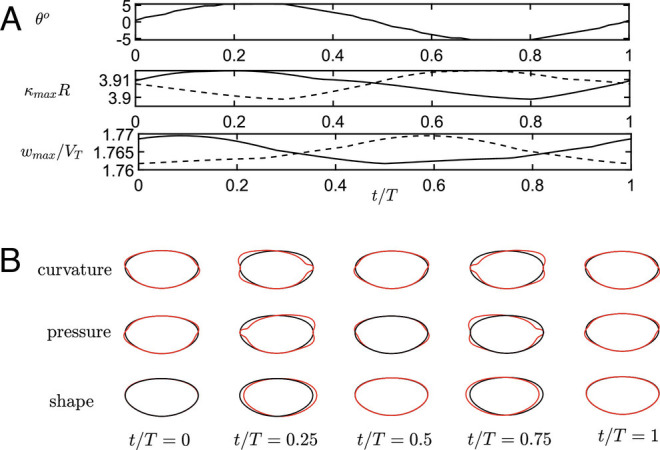
Periodic perturbations during zigzag motion, the amplitude of the linear perturbation having been chosen arbitrarily. (*A*) Tilt angle *θ*, maximum curvature, and maximum axial velocity, as function of time. Solid lines: right side of the drop, dashed lines: left side. (*B*) Snapshots of the perturbations to curvature, pressure, and bubble shape are plotted on the bubble surface. The unperturbed bubble is shown in black, and the perturbation is plotted in red in the normal direction on each point on the surface.

In Fig. 2, we characterize the periodic instability by the interplay of flow and surface perturbations, similar to arguments proposed in ref. (9). This motion is slow compared to the time scale on which the bubble is rising, as characterized by the Strouhal number *St* = 2*f* *R*/*V*_*t*_ = 0.032 being small. As seen in Fig. 2*A*, *Top* the bubble undergoes a periodic tilt, with one side pointing up being correlated with a higher curvature on the same side (second panel). Greater curvature implies greater surface vorticity, increasing the rise velocity (third panel from *Top*). The differences in rise velocity then (with some phase shift) translate into a tilt, the side with the greater curvature pointing up.

In Fig. 2*B*, the distribution of curvature, pressure, and deformation over the surface of the bubble are shown in greater detail. The *Top* row shows the curvature, a large value of which makes the surface more “slippery,” so that the fluid moves faster. Then, by the Bernoulli theorem, the pressure is lower where the fluid moves faster, as seen in the second row, and the imbalance in pressure pushes back the bubble to its original position, as seen in the last row. This also reverses the distribution of curvature, and the process repeats itself.

In conclusion, we have found a mechanism for the periodic motion of a rising bubble, qualitatively different from the behavior of a solid particle. This opens the door to the study of small contaminations, present in most practical settings, which emulate a particle somewhere in between a solid and a gas.

## Supplementary Material

Appendix 01 (PDF)

## Data Availability

Data for figures is available at https://zenodo.org/record/7342806. All study data are included in the article and/or *SI Appendix*.
