# Supplementary material for: Path instability of an air bubble rising in water
Source: Proc Natl Acad Sci U S A. 2023 Jan 17;120(4):e2216830120. doi: 10.1073/pnas.2216830120 (PMC9942867; doi:10.1073/pnas.2216830120)
Supplement: Supplementary file 1 — Appendix 01 (PDF) [file pnas.2216830120.sapp.pdf]

Supporting Information for

Path instability of an air bubble rising in water

Miguel A. Herrada, Jens G. Eggers

Jens G. Eggers.
E-mail: jens.eggers@bristol.ac.uk

This PDF file includes:

Supporting text
SI References

Supporting Information Text

Formulation

We consider the configuration where a bubble of a gas of density ρ_g and viscosity μ_g is released into a large reservoir of liquid of density ρ_l and viscosity μ_l. To model the rise of the bubble under the action of gravity g, a non-inertial cylindrical coordinate system $(z,\, r,\, \theta)$ aligned with the gravity vector and anchored to the bubble is selected. This system is in turn linked to an inertial Cartesian coordinate system $(Z,\, X,\, Y)$, also aligned with the gravity field and the non-inertial system. To model the apparent forces in the momentum equations in this non-inertial coordinate system, we have to include all the accelerations of the system. In our case, since both coordinate systems are aligned, we will only have to consider the axial acceleration $\mathbf{a} = dV/dt\mathbf{e}_Z$ of the reference frame, where here $V(t)$ is the axial velocity of the top point of the liquid-gas interface in the inertial system. The computational domain used in the simulations is a sphere of radius R_{out} much larger than the effective bubble radius.

A. Equations of motion. The conservation of mass and momentum in the gas $(i = l)$ and liquid $(i = g)$ subdomains is given by

$$\boldsymbol{\nabla}\cdot\mathbf{v}_i = 0, \qquad (i = l, g), \qquad [1a]$$

$$\rho_i\left(\frac{\partial\mathbf{v}_i}{\partial t} + (\mathbf{v}_i\cdot\boldsymbol{\nabla})\,\mathbf{v}_i\right) = \boldsymbol{\nabla}\cdot\boldsymbol{\sigma}_i + \rho_i\left(g + \frac{dV}{dt}\right)\mathbf{e}_z, \qquad (i = l, g), \qquad [1b]$$

where $\mathbf{v}_i = w_i\mathbf{e}_z + u_i\mathbf{e}_r + v_i\mathbf{e}_\theta$ is the velocity field and $\boldsymbol{\sigma}_i$ is the stress tensor of material i $(i = g, l)$. Each stress tensor depends on the characteristics of the material through a constitutive model. We consider in both regions an incompressible Newtonian fluid, where this stress tensor takes the form

$$\boldsymbol{\sigma}_i = -p_i\mathbf{I} + \mu_i\left(\boldsymbol{\nabla}\mathbf{v}_i + \boldsymbol{\nabla}\mathbf{v}_i^T\right), \qquad (i = l, g) \qquad [1c]$$

p_i $(i = l, g)$ is the pressure.

Upstream of the bubble, at $r = R_{out}$ and $z > 0$ we assume that in the inertial system, the flow is at rest:

$$w_l = -V(t), \quad u_l = 0, \quad v_l = 0, \quad p_l + \rho_l\left(g + \frac{dV}{dt}\right)z = 0. \qquad [1d]$$

Downstream of the bubble, at $r = R_{out}$ and $z \leq 0$ we need to relax these boundary conditions in order to capture the wake. The following non-reflecting boundary conditions have been applied

$$\rho_l\frac{\partial w_l}{\partial t} + \rho_l w_l\frac{\partial w_l}{\partial z} = \rho_l\left(g + \frac{dV}{dt}\right), \quad \rho_l\frac{\partial u_l}{\partial t} + \rho_l w_l\frac{\partial u_l}{\partial z} = 0, \quad \rho_l\frac{\partial v_l}{\partial t} + \rho_l w_l\frac{\partial v_l}{\partial z} = 0. \qquad [1e]$$

Across the interface between liquid and gas, which is parameterized in terms of a meridional arc length s $(0 \leq s \leq 1)$ and the azimuthal angle θ, $r_I = f(s,\theta,t)$ and $z_I = h(s,\theta,t)$, we impose that the velocity field must be continuous, in the form

$$w_l = w_g, \quad u_l = u_g, \quad v_l = v_g. \qquad [1f]$$

We also impose a balance of normal and tangential stresses between the liquid and the gas, in the form

$$\mathbf{n}\cdot(\boldsymbol{\sigma}_l - \boldsymbol{\sigma}_g)\cdot\mathbf{n} = \gamma\kappa, \qquad [1g]$$

$$\mathbf{t}_1\cdot(\boldsymbol{\sigma}_l - \boldsymbol{\sigma}_g)\cdot\mathbf{n} = 0, \quad \mathbf{t}_2\cdot(\boldsymbol{\sigma}_l - \boldsymbol{\sigma}_g)\cdot\mathbf{n} = 0, \qquad [1h]$$

where

$$\mathbf{n} = \frac{h_s\mathbf{e}_r - f_s\mathbf{e}_z + (f_s h_\theta - f_\theta h_\theta)/f\mathbf{e}_\theta}{[h_s^2 + f_s^2 + ((f_s h_\theta - f_\theta h_\theta)/f)^2]^{1/2}}, \quad \mathbf{t}_1 = \frac{h_s\mathbf{e}_z + f_s\mathbf{e}_r}{(f_s^2 + h_s^2)^{1/2}}, \quad \mathbf{t}_2 = \mathbf{n}\times\mathbf{t}_1$$

are normal $(\mathbf{n})$ and tangential vectors $(\mathbf{t})$ to the surface, the subscripts s and θ represent derivatives with respect to s and θ respectively, $\kappa = \nabla\cdot\mathbf{n}$ is (twice) the mean curvature and γ the surface tension. On the other hand, the kinematic boundary condition becomes

$$\left(u_l - \frac{\partial f}{\partial t}\right)\frac{\partial h}{\partial s} - \left(w_l - \frac{\partial h}{\partial t}\right)\frac{\partial f}{\partial s} + \frac{v_l}{f}\left(\frac{\partial f}{\partial s}\frac{\partial h}{\partial \theta} - \frac{\partial f}{\partial \theta}\frac{\partial h}{\partial s}\right) = 0. \qquad [1i]$$

To ensure a uniform distribution of points along the interface coordinate s, the following additional equation is imposed:

$$\frac{\partial f}{\partial s}\frac{\partial^2 f}{\partial s^2} + \frac{\partial h}{\partial s}\frac{\partial^2 h}{\partial s^2} = 0. \qquad [1j]$$

On the axis, $r = 0$, regularity conditions as given in (1) are imposed.

For unsteady 3D simulations, the kinematic equation Eq. (1i) and the continuity equations Eq. (1a) guarantee that the volume of the bubble, V_o, remains constant. However, for axisymmetric simulations where large time steps are used, to reach the steady state and to keep the volume unchanged the following additional equation must be imposed

$$V_o = \int_0^1 \pi f^2\frac{\partial h}{\partial s}ds. \qquad [1k]$$

Finally, to close the problem we need an additional condition for the rising velocity V. This is achieved by imposing that a point of the interface to remain fixed in the non-inertial reference frame. We have chosen the upper point of the interface that crosses the axis where the following equation is verified

$$w_l = 0, \quad z_I = h = R, \quad r_I = f = 0, \quad at \quad s = 1, \qquad [11]$$

where $R = (3/4V_o/\pi)^{1/3}$ is the undisturbed radius.

B. Mapping technique. The spatial domain occupied by gas is mapped onto a rectangular domain by means of a non singular mapping

$$r = f_g(s, \eta_g, \theta, t), \quad z = h_g(s, \eta_g, \theta, t), \quad [0 \le s \le 1] \times [0 \le \eta_g \le 1],$$

where the shape functions f_g and h_g are obtained as a part of the solution. In order to capture large anisotropic deformations, the following quasi-elliptic transformation (2) was applied

$$g_{22}\frac{\partial^2 f_g}{\partial s^2} + g_{11}\frac{\partial^2 f_g}{\partial \eta_g^2} - 2g_{12}\frac{\partial^2 f_g}{\partial s \partial \eta_g} = Q, \qquad [2a]$$

$$g_{22}\frac{\partial^2 g_g}{\partial s^2} + g_{11}\frac{\partial^2 h_g}{\partial \eta_g^2} - 2g_{12}\frac{\partial^2 h_g}{\partial s \partial \eta_g} = 0, \qquad [2b]$$

where the coefficients take the form

$$g_{11} = \left(\frac{\partial h_g}{\partial s}\right)^2 + \left(\frac{\partial f_g}{\partial s}\right)^2, \quad g_{22} = \left(\frac{\partial h_g}{\partial \eta_g}\right)^2 + \left(\frac{\partial f_g}{\partial \eta_g}\right)^2, \quad g_{12} = \frac{\partial h_g}{\partial \eta_g}\frac{\partial h_g}{\partial s} + \frac{\partial f_g}{\partial \eta_g}\frac{\partial f_g}{\partial s},$$

with

$$Q = -\left(\frac{\partial D_1}{\partial \eta_g}\frac{\partial f_g}{\partial s} - \frac{\partial D_1}{\partial s}\frac{\partial f_g}{\partial \eta_g}\right)\frac{J}{D_1}, \quad J = \frac{\partial h_g}{\partial \eta_g}\frac{\partial f_g}{\partial s} - \frac{\partial h_g}{\partial s}\frac{\partial f_g}{\partial \eta_g},$$

and

$$D_1 = \epsilon_p \sqrt{\left[\left(\frac{\partial f_g}{\partial s}\right)^2 + \left(\frac{\partial h_g}{\partial s}\right)^2\right] / \left[\left(\frac{\partial f_g}{\partial \eta_g}\right)^2 + \left(\frac{\partial h_g}{\partial \eta_g}\right)^2\right]} + (1 - \epsilon_p).$$

In the above expressions, ϵ_p is a free parameter between 0 and 1 where the case $\epsilon_p = 0$ corresponds to the classical elliptical transformation. All the simulations in this work were conducted using $\epsilon_p = 0$.

Some additional boundary conditions for the shape functions are needed to close the problem.

At the free surface, located at $\eta_g = 1$,

$$f_g(s, \eta_g, \theta, t) = f(s, \theta, t), \quad h_g(s, \eta_g, \theta, t) = h(s, \theta, t). \qquad [3]$$

At the axis, located at $\eta_g = 0$

$$f_g(s, \eta_g, \theta, t) = 0, \quad \frac{\partial h_g}{\partial \eta_g} = 0. \qquad [4]$$

The spatial domain occupied by the liquid is also mapped into a rectangular domain by means of an analytical mapping in the form

$$r = f(s, \theta, t) + [R_{out}\sin(\pi s) - f(s, \theta, t)]\eta, \qquad [5]$$

$$z = h(s, \theta, t) + [R_{out}\cos(\pi s) - h(s, \theta, t)]\eta, \quad [0 \le s \le 1] \times [0 \le \eta \le 1]. \qquad [6]$$

The unknown variables in the liquid domain are w_l, u_l, v_l, p_l f, h and V while the unknown variables in the gas domain are f_g, h_g, w_g, u_g, u_g and p_g, and all the derivatives appearing in the governing equations are expressed in terms of s, η_g, η, θ and t. These mappings are applied to the governing equations Eq. (1) and the resulting equations are discretised in the η-direction with n_η and n_{η_g} Chebyshev spectral collocation points in the liquid and gas domains, respectively. In the liquid domain an additional stretching function was used to concentrate the points near the bubble surface ($\eta_{lstretch} = \tanh[1 - \eta]/\tanh(1)$). Conversely, in the s-direction we use second-order finite differences with n_s equally spaced points. To compute the basic axisymmetric flows we will remove all θ derivatives from the system, while a classical modal decomposition will be used to compute the θ derivatives in the perturbed 3D flow. The results presented in this work were carried out using $n_s = 301$, $n_{\eta_g} = 11$ and $n_\eta = 91$. We checked that the results presented in this work do not depend on the mesh.

C. Non Linear Axysimmetric Steady solutions. Steady solutions of the nonlinear equations Eq. (1) with all independent variables of θ are obtained by pseudo time integration of these equations. The time derivatives are discretised using backward second order differences and at each time step the resulting system of (nonlinear algebraic) equations is solved using the Newton-Raphson technique. Since we are only interested in the final steady configuration and the scheme is fully implicit, larger time steps are used to reach these steady solutions. As initial condition for the simulation, a perfect spherical bubble of small radius with the flow at rest is considered. Once the solution has converged to a steady state, it is used as an approximate solution in Newton's procedure and the bubble radius is increased. If the solution does not converge using a single time step, the time step is reduced and the equations are integrated until a new steady solution is reached.

D. Small amplitude 3D perturbations. To test the stability of a given steady state we calculate the linear three-dimensional global modes by assuming the temporal and azimuthal dependences

$$\Psi(z, r, \theta; t) = \Psi_b(z, r) + \epsilon\, \delta\Psi(z, r) e^{-i\omega t + im\theta}, \quad (\epsilon \ll 1), \tag{7}$$

where $\Psi(z, r, \theta; t)$ represents any dependent variable while $\Psi_b(z, r)$ and $\delta\Psi(r, z)$ denote the base (steady) solution and the spatial dependence of the eigenmode for that variable, respectively, while $\omega = \omega_r + i\omega_i$ is the frequency (an eigenvalue) and m is the azimuthal wave number. For a given m, both the eigenfrequencies and the corresponding eigenmodes are calculated as a function of the governing parameters. The dominant eigenmode is that with the largest growth factor ω_i. If that growth factor is positive, the base flow is asymptotically unstable.

The numerical procedure used to solve the steady problem can be easily adapted (3) to solve the eigenvalue problem which determines the linear global modes of the system. In this case, the temporal and azimuthal derivatives are computed assuming the dependence Eq. (7). The spatial dependence of the linear perturbation $\delta\Psi^{(q)}$ is the solution to the generalized eigenvalue problem $\mathcal{J}_b^{(p,q)} \delta\Psi^{(q)} = i\omega \mathcal{Q}_b^{(p)} \delta\Psi^{(q)}$, where $\mathcal{J}_b^{(p,q)}$ is the Jacobian of the system evaluated with the basic solution $\Psi_b^{(q)}$, and $\mathcal{Q}_b^{(p,q)}$ accounts for the temporal dependence of the problem. This generalized eigenvalue problem is solved using MATLAB `eigs` function.

E. Control parameters. To non-dimensionalise the system we used the radius of the bubble R, the surface tension γ and the liquid density ρ_l . The resulting problem is governed by four dimensionless parameters,

$$Oh = \frac{\eta_l}{\sqrt{\rho_l R^3 \gamma}}, \quad Bo = \frac{\rho g R^2}{\gamma}, \quad \rho = \frac{\rho_g}{\rho_l}, \quad \mu = \frac{\mu_g}{\mu_l}. \tag{8}$$

Oh is the Ohnesorge number, Bo is the Bond number, while ρ and μ are the density and viscosity ratio respectively.

We are interested in analyzing the bubble terminal velocity and the global 3D stability of the axisymmetric base flow around the bubble as a function of the initial bubble radius for a given liquid-gas configuration. Therefore, knowing the properties of water and air at 19.6^{o}C (4) gives $\rho_l = 998.26\ kg/m^3$, $\mu_l = 1.0142$ mPa · s, $\rho_g = 1.225\ kg/m^3$, $\mu_g = 1.810^{-5}$ Pa · s and $\gamma = 72.8\ mN/m$ (5). Equation (8) is then used to calculate the results as a function of R. In the simulations, the outer radius of the computational domain was kept fixed, $\hat{R} = \frac{R_{out}}{R} = 15$.

References

1. MA Herrada, C Ferrera, JM Montanero, AM Gañán-Calvo, Absolute lateral instability in capillary coflowing jets. *Phys. Fluids* **22**, 064104 (2010).
2. Y Dimakopoulos, J Tsamopoulos, A quasi-elliptic transformation for moving boundary problems with large anisotropic deformations. *J. Comput. Phys.* **192**, 494–522 (2003).
3. MA Herrada, JM Montanero, A numerical method to study the dynamics of capillary fluid systems. *J. Comp. Phys.* **306**, 137–147 (2016).
4. PC Duineveld, The Rise Velocity and Shape of Bubbles in Pure Water at High Reynolds Number. *J. Fluid Mech.* **292**, 325–332 (1995).
5. DR Lide (ed.), *Handbook of Chemistry and Physics.* (CRC Press), (2003).
